# Differences in phenology across three trophic levels between two Afrotropical sites separated by four degrees latitude

**DOI:** 10.1002/ece3.70274

**Published:** 2024-09-11

**Authors:** Crinan Jarrett, Ojodomo Godday Simon, Christian N. Tchana, Thaddeus Apezan Pev, Michelle Fany Meigang Kamkeng, Alain Christel Wandji, Shiiwua A. Manu, Mélanie Adèle Tchoumbou, Barbara Helm, Luke L. Powell, Chima J. Nwaogu

**Affiliations:** ^1^ Swiss Ornithological Institute Bird Migration Unit Sempach Switzerland; ^2^ School of Biodiversity, One Health and Veterinary Medicine, College of Medical, Veterinary and Life Sciences University of Glasgow Glasgow UK; ^3^ Biodiversity Initiative Houghton Michigan USA; ^4^ A.P. Leventis Ornithological Research Institute Jos Nigeria; ^5^ Department of Biology‐Ecology, Faculty of Sciences University of Montpellier Montpellier France; ^6^ Department of Animal Biology and Physiology, Faculty of Sciences University of Yaoundé 1 Yaounde Cameroon; ^7^ Šivickis Laboratory of Parasitology Nature Research Center Vilnius Lithuania; ^8^ CIBIO‐InBIO, Research Centre in Biodiversity and Genetic Resources University of Porto Vairão Portugal; ^9^ BIOPOLIS Program in Genomics, Biodiversity and Land Planning CIBIO Vairão Portugal; ^10^ FitzPatrick Institute of African Ornithology, DST‐NRF Centre of Excellence University of Cape Town Cape Town South Africa

**Keywords:** arthropod, birds, breeding, common bulbul, fruit tree, moult, seasonality

## Abstract

Birds time their life cycle events to favourable windows in environmental conditions. In tropical environments, where photoperiod variation is small, birds show high variability in the timing of life cycle stages, yet these species have been severely underrepresented in phenology research. Here, we investigated temporal patterns in bird life cycles and resource availability in two sites in tropical Africa: Weppa (Nigeria, 7° N) and Elat (Cameroon, 3° N). In these sites we captured common bulbuls (*Pycnonotus barbatus*), a widespread generalist, and recorded breeding and moult over a 12‐month period. Simultaneously, we surveyed fruiting tree and arthropod abundance. Our aim was to quantify seasonal patterns in moult and breeding in bulbuls at both sites, and link them to fluctuations in local fruit and arthropod abundance and precipitation. Moult was more seasonal than breeding in both sites, and seasonality of both life cycle events was stronger in Nigeria than Cameroon. The peak timing for moult was 1.5 months earlier in Nigeria than Cameroon. Seasonal variation in abundance of fruiting trees and arthropods was different between sites, as were the associations with breeding and moulting. In Nigeria, we found a positive association between moult and arthropod abundance, and a negative one with fruiting tree abundance. In contrast, in Cameroon moult was associated with higher precipitation, while breeding occurred at times with higher fruit abundance. Our results provide evidence that, even in similar habitats separated by four degrees in latitude, seasonal patterns across three trophic levels are variable. Understanding links between environmental conditions and life cycle events can reveal potential vulnerabilities of tropical species, and guide conservation efforts.

## Graphical abstract

In tropical environments, where photoperiod variation is small, birds show high variability in the timing of life cycle stages, yet these species have been severely underrepresented in phenology research. We investigated temporal patterns in breeding and moult in an Afrotropical bird, finding that moult was more seasonal than breeding. Additionally, we found that timing of these life‐cycle events, and seasonality in lower trophic levels (arthropods, plants) were different between two tropical sites separated by 4 degrees latitutde.

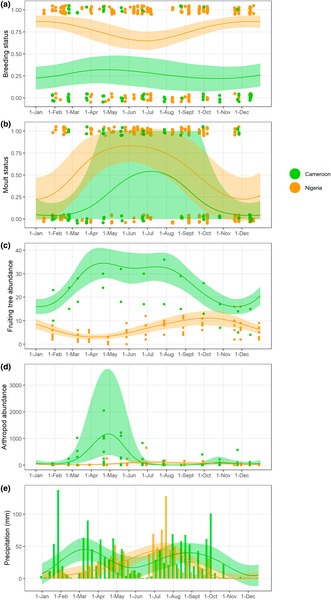

## 1 INTRODUCTION

To maximise fitness, organisms time life cycle events to coincide with windows of favourable environmental conditions. For birds, such events include breeding and moulting which, in temperate regions, are timed to follow the predictable seasonal rise in food availability in spring arising from a strong yearly oscillation in photoperiod and temperature. Temporal mismatch between life cycle events and environmental resources has considerable costs for birds in these regions (Visser & Gienapp, 2019). In tropical areas, where photoperiod and temperature oscillate less strongly, the timing of life cycle events in birds can be much more variable (Jenni & Winkler, 2020; Nwaogu et al., 2019). Our understanding of how tropical birds time their life cycle events in relation to environmental conditions has improved over recent years but remains severely biased by the relative volume of research from temperate regions (Cohen et al., 2018; Grames et al., 2023). Given that most the world's bird species reside in the tropics, this is a bias that must be addressed to allow a more objective understanding of biological rhythms in birds.

A potential consequence of the temperate bias in avian life cycle studies is a focus on breeding as the most critically timed event in the life cycle. Breeding must be well timed because it is an energy expensive process that requires high food availability both to maintain the parents' body conditions and to feed chicks. In temperate birds, where the window of suitable breeding conditions occurs once per year, and where longevity is thought to be in general low (Hau et al., 2010), it is assumed that maximising breeding success through accurate synchronisation to food resources is under strong selection pressure. However, in tropical regions, priorities may be different; birds tend to live longer, and nest predation is often higher, and therefore fitness may be strongly mediated through survival, as bird must survive until the next breeding attempt (Class & Moore, 2013; Jetz et al., 2008; Martin et al., 2017; Muñoz et al., 2018; Stevens et al., 2013). To increase survival, birds may therefore prioritise self‐maintenance processes such as moult (Class & Moore, 2013; Jenni & Winkler, 2020).

Moult can be a costly life cycle event, due to the direct energetic cost of regrowing feathers and accompanying tissues, as well as additional costs such as increased thermoregulation and less effective predator avoidance during this period (Jenni & Winkler, 2020). For successful moult, birds must have access to sufficient food, including certain important nutrients (Jenni & Winkler, 2020). Food limitation, or lack of nutrients such as protein, carotenoids, or certain amino‐acids, can lead to depletion of body reserves, slower moult or the production of lower quality feathers (Hill, 2000; Murphy et al., 1988; Swaddle & Witter, 1997). To increase the chance of surviving until the next breeding attempt, it is logical that tropical species may synchronise moult, more than breeding, to favourable environmental conditions. However, so far, we know little about which environmental variables may be associated to higher probability of moulting in the tropics, and few studies have tested whether moult is indeed more seasonal than breeding in these environments (Class & Moore, 2013; Jenni & Winkler, 2020; Nwaogu et al., 2019).

One aspect that makes studying tropical bird life cycles complicated is the extent of variation between individuals, populations, regions and years. This has led to the impression that tropical species may lack seasonality (Baker, 1939). However, evidence from tropical regions demonstrates that species often do show seasonal life cycles that are distinct between populations even in close spatial proximity. One extreme example is a ~5 months difference in the timing of breeding of two populations of rufous‐collared sparrows (*Zonotrichia capensis*) separated by 25 km (Moore et al., 2005). This stark difference is explained by shifted weather patterns between the sites given their position on either side of the Andean divide. A similar phenomenon occurs in Cameroon, where highland populations of the same species breed in a different season to lowland populations, possibly as a consequence of rainfall patterns (Tye, 1992). Some other studies from the tropics and subtropics show that timing of breeding and moult entrain with local rainfall patterns along environmental gradients (Nwaogu & Cresswell, 2020; Oschadleus & Underhill, 2006). These studies demonstrate that when tropical species are studied at broad spatial scales, local seasonal variation may be masked resulting in a deceptively non‐seasonal pattern in life cycle events (Baker, 1939). Additionally, they demonstrate that seasonality in weather conditions, and consequently in lower trophic levels such as plants and insects, may also vary over small spatial scales. Overall, these studies suggest locally adapted timing of breeding and moulting of birds in the tropics—but offer little insight into the actual environmental factors that could influence such timings, aside from rainfall.

Thus, for a more comprehensive understanding of avian seasonality, data from tropical species are needed that link breeding and moult to fluctuations in key aspects of the environment, such as climate and underlying trophic levels (Visser & Gienapp, 2019). In temperate areas, where phenology of breeding and moult is associated with the yearly raising temperatures and longer days of spring, studies tend to investigate inter‐annual variation in timing and its drivers. However, in tropical areas, breeding and moult may be spread throughout the year, and so it becomes natural to investigate intra‐annual drivers of phenology. This is especially true given the paucity of tropical phenology data available, which is partly explained by the challenges of funding and logistics for collecting year‐round data (Cohen et al., 2018). To help fill this gap, in this study, we provide comparative annual‐cycle data on three trophic levels, recorded for one full year. The logistical trade‐off we faced, given the demands of detailed tri‐trophic coverage, meant we could only collect data simultaneously at two sites and in a single year. We cannot therefore make conclusions about the repeatability of observed patterns, but we are able to investigate relationships between short‐term temporal variation in the environment and bird life cycles, and assess whether these patterns are consistent between sites.

We build on an avian system introduced by Nwaogu et al. (2019), in which the target species, the common bulbul (*Pycnonotus barbatus*; hereafter called “bulbul”), showed strong annual cycles in moult and weak cycles in breeding near Jos in Nigeria (9°52′ N, 08°58′ E). The common bulbul is a good model species for phenological study. It is a common and relatively easily observed year‐round resident in the study region, while also being widespread throughout the African continent. Therefore, it faces very distinct conditions throughout its range and likely shows local variation in life cycle timing (Jenni & Winkler, 2020; Nwaogu & Cresswell, 2020). Bulbuls are omnivorous birds and may therefore meet the nutritional requirements of moult and breeding with different combinations of plant and arthropod foods, according to local conditions. They are known to consume a wide range of plant and arthropod taxa, including termites, ants, caterpillars, butterflies, mango, guava, African plum, and many other fruits (CJ, CJN & AJW, unpublished data; Nwaogu, 2019). Therefore, phenological variation at lower trophic levels can be assumed to be roughly reflected in the bulbuls' diet. For a more direct description of trophic relationships of bulbuls, we have recently used faecal metabarcoding to assess diet around the year at the study site in Cameroon (CJ, CJN & AJW, unpublished data). This study confirmed that bulbuls consume a wide range of plant and arthropod taxa, broadly overlapping with the taxa surveyed in this study. However, moulting bulbuls had a more diverse diet than non‐moulting birds, both in terms of fruit and arthropods, leading to the hypothesis that bulbuls may synchronise moult to periods of plentiful resources (CJ, CJN & AJW, unpublished data). In contrast, a captive study on bulbuls showed that a fruit‐only diet instigated earlier moult compared with an arthropod‐only diet (Nwaogu et al., 2020). Nwaogu and Cresswell (2020) assessed moult along a latitude gradient in Nigeria, and found that with increasing latitude in Nigeria, moult occurs later, matching the pattern in the onset of rains. The onset of rains may in turn drive the emergence of plant and insect resources, exploited by bulbuls during moult. Based on these studies, it seems that bulbuls seek out a diverse diet during moult (perhaps with high fruit content), and these foods may become available after the onset of the rains. However, we still lack data from the wild that links timing of moult and breeding in bulbuls to fluctuations in environmental resources.

Our aim was to test three main predictions: first, we expected breeding and moult to show seasonality at both sites, with moult showing a stronger seasonal signal than breeding (Class & Moore, 2013; Nwaogu et al., 2019). Second, we expected to see parallel phenology between sites, with earlier timings in Cameroon due to the earlier onset of the rains (Nwaogu & Cresswell, 2020), and with links between timing of moulting and fruit availability, following Nwaogu et al. (2020), and between timing of breeding and insect availability, following literature from temperate environments (Perrins, 1991; Visser & Gienapp, 2019). Finally, we expected the site closer to the equator to show weaker seasonality than the northern site, though with only two sites in the comparison this contrast cannot be conclusively attributed to latitudinal effects. As our aims involved linking differences in timing of breeding and moult of bulbuls to environmental phenology, we chose to focus on arthropods, fruit and precipitation as representatives of food availability in the environment.

## 2 METHODS

### 2.1 Study sites and local precipitation patterns

The data for this study were collected at two sites in the Afrotropics: Weppa Farm (Nigeria: 7°2′1 N, 6°35′31 E) and Elat Farm (Cameroon: 3°54′12″ N, 11°42′59″ E; Appendix S1). For simplicity, we will henceforth use Nigeria and Cameroon to refer to Weppa and Elat respectively. The habitat at both sites was a mix of natural vegetation and agricultural land, in Nigeria consisting in a mix of crops including oil palm, cashew, cassava and maize, and in Cameroon in cocoa agroforestry (Ivande, 2015; Jarrett et al., 2021). Approximately once per month between March 2021 and March 2022 (for precise dates of visits, see Appendix S1), field teams conducted fruit tree and arthropod surveys, with the aim to quantify proxies of potential food availability for bulbuls. Bird surveys were conducted on several days per month at each site (Appendix S1). For information on precipitation, we used the reanalysis product MERRA‐2, which includes corrected daily precipitation measures (Rienecker et al., 2011). We downloaded the datasets corresponding to the sampling period of March 2021 to March 2022 for both sites from the National Aeronautics and Space Administration Langley Research Center Prediction of Worldwide Energy Resource Project (https://gmao.gsfc.nasa.gov/reanalysis/MERRA‐2/).

### 2.2 Bird data collection

During each visit, we captured common bulbuls, a resident, generalist species, using mist‐nets. Depending on the local conditions and knowledge of areas preferred by bulbuls, between 10 and 14 mist‐nets (12 m long) were set up at dawn, and left open for ~6 h. We checked nets every 20 min, and released any captured birds that were not bulbuls at the net. All bulbuls were brought back to the processing station, where they were ringed. Then, we scored brood patch using EURING standard codes (EURING, 2010), and cloacal protuberance as ‘S’, ‘M’ or ‘L’, where ‘S’ meant no obvious swelling of cloaca, ‘M’ meant obvious swelling with columnar shape, and ‘L’ meant considerable swelling of cloaca with bulbous shape. For subsequent analyses, we classified birds into two categories: actively breeding or not actively breeding. We considered male birds with a cloacal protuberance score of ‘M’ and ‘L’ and female birds with brood patch score 1–4 as actively breeding, and all other individuals as not actively breeding.

Primary moult was scored using the EURING system, where each of the 10 primary feathers of the right wing is given a score between 0 and 5 (EURING, 2010). A score of 0 indicates an old non‐moulted feather, scores 1–4 indicate progressively growing feathers, and 5 is a newly grown feather. Then, we added up the score across the 10 primaries to give a total moult score between 0 (all old feathers) and 50 (all new feathers). We then categorised birds as either performing primary moult (primary moult score >0 and <50) or not (primary moult score 0 or 50), thus converting moult to a binary variable, as with breeding.

### 2.3 Fruit surveys

In each site we established a series of 100 m transects, placed at least 10 m from the edge of the agricultural habitat. In the Cameroonian site there were 2 transects, and in the Nigerian site 5, due to the larger area of the latter. During each visit, we walked these transects and counted the number of trees that were visibly fruiting. Trees were identified to species level where possible, or to genus level. The resulting dataset consisted in the number of fruiting trees of each species or genus on each transect.

### 2.4 Arthropod surveys

At each site, we selected four trees on which to conduct arthropod surveys. In Nigeria, we chose 2 *Bauhinia thonningii* individuals, one *Ficus sur* and one *Nauclea latifolia*. In Cameroon, all four chosen trees were *Theobroma cacao*. The tree species were selected to be representative of the local environment in which the birds forage. During each visit, we surveyed all arthropods on these trees using a combination of visual surveys, hand‐netting (to capture flying individuals) and branch beating (Montgomery et al., 2021). All arthropods collected were placed into falcon tubes containing 80% ETOH, and then counted and identified to the lowest possible taxonomic level in the laboratory upon returning from the field. For subsequent analyses, we considered the sum of individuals across survey methods as they mainly targeted different taxa.

### 2.5 Data analyses

#### 2.5.1 Testing for seasonality across trophic layers

Overall, as our interest was in observing seasonal patterns, we used day in the year, where 1 represents 1‐January, and 365 represents 31‐December. To assess seasonal patterns in all trophic levels (birds, arthropods, fruit), we used generalised additive models (GAMs) using package mgcv (Wood, 2017) with a smoothed term for day in the year as explanatory variable, with a cyclic cubic spline. Previous studies have demonstrated that when investigating long‐term trends in data containing temporal replicates (e.g., multiple counts each month), it is necessary to also include a random effect for time to account for temporally replicated data points (Daskalova et al., 2021; Knape, 2016). This random effect should be at the temporal scale that captures repeated visits (e.g., year, if multiple surveys are made per year). To allow variation to be partitioned between the smoothed term and the random effect in the GAMs, the degrees of freedom in the smoothed term must be restricted; otherwise, all variance is absorbed by this term. Here, we restricted the degrees of freedom used by the smooth term to 4, following the rule proposed by Knape (2016) in which degrees of freedom are restricted to one third the number of survey times (here 12—once monthly for a year).

We used week of the year (numeric with potential range 1–52) as a random factor in our models. We used week of the year instead of month because in a few instances in Nigeria, counts were not spaced out exactly monthly (e.g., 3‐March and 30‐March; see Appendix S1), meaning that if we used month as random factor the same intercept would be imposed on these temporally separated counts. For counts that are spaced out monthly, week of the year results in exactly the same use of degrees of freedom in the models as monthly (i.e., week 1, 5, 10, etc rather than month 1, 2, 3 …; Appendix S1).

To investigate seasonality in breeding and moulting in birds, our response variables were breeding or moulting status (1 or 0), and we assumed a Bernoulli distribution. To investigate seasonality in fruiting tree abundance, for each site we built a GAM with fruiting tree abundance per transect as response variable, assuming a Poisson distribution. We included the temporal smoothed term and random effect and a fixed effect for transect. For arthropod abundance we built a similar GAM for each site but including a fixed effect for tree rather than transect. We tested for over or under‐dispersion in our Poisson models using the package DHARMa (Hartig, 2022), and when found switched to negative binomial distribution. In one model (arthropod abundance in Cameroon), there were issues with overdispersion both in the Poisson and Negative binomial, so instead we log‐transformed arthropod count data and modelled as Gaussian. In all cases, we tested for presence of seasonality by comparing the model with the smoothed temporal term to a model without it (i.e., including just the random temporal term and any additional terms such as transect or tree; Table 1).

**TABLE 1:** Results from Generalised additive models (GAMs) and circular statistics to describe variation in timing of breeding and moulting, fruit tree and arthropod abundance in Nigeria and Cameroon.

Site	Response variable	Generalised additive models (GAMs)				Circular statistics		
		Explanatory variables	*R* ^2^	AIC	∆AIC	Circular mean	Circular mode	Rayleigh test *p*‐value
Nigeria	Breeding	Day of year + (1|week)	.05	260.0	0.0	2.19 (02–01)	7.05 (07–01)	.37
		(1|week)	.06	265.6	5.6			
	Moulting	Day of year + (1|week)	.25	286.8	0.0	169.92 (21–06)	166.97 (18–06)	<.01
		(1|week)	.25	294.1	7.3			
	Fruit tree abundance	Day of year + (1|week) + transect	.38	297.4	0.0	273.85 (30–09)	273.35 (30–09)	<.01
		(1|week) + transect	.35	301.6	4.2			
	Arthropod abundance	Day of year + (1|week) + tree	.60	465.1	0.0	201.19 (18–07)	190.22 (12–07)	<.01
		(1|week) + tree	.61	467.3	2.2			
	Precipitation	Day of year	.43	467.3	0.0			
		1	.00	493.5	26.3			
Cameroon	Breeding	Day of year + (1|week)	.02	75.2	0.0	148.35 (30–05)	118.36 (30–04)	.11
		(1|week)	.01	75.4	0.2			
	Moulting	Day of year + (1|week)	.23	77.7	0.0	198.80 (16–07)	193.03 (15–07)	<.01
		(1|week)	.22	79.2	1.5			
	Fruit tree abundance	Day of the year + (1|week) + transect	.86	139.4	0.0	159.43 (11–06)	159.22 (10–06)	<.01
		(1|week) + transect	.88	146.0	6.6			
	log(Arthropod abundance)	Day of year + (1|week) + tree	.52	179.1	0.0	125.80 (08–05)	126.11 (08–05)	<.01
		(1|week) + tree	.50	185.0	5.9			
	Precipitation	Day of year	.16	509.8	0.0			
		1	.00	515.8	6.0			

*Note*: AIC and *R*‐squared values for GAMs including the smoothed seasonal term vs null model are shown, as well as the difference in AIC between models (∆AIC). For circular statistics, we include circular mean, circular mode, and the results from the Rayleigh test which tests for uniformity (i.e., non‐seasonality) of distribution.

We compared models using Akaike's Information Criterion (AIC), and considered that there was evidence for seasonality when the difference in AIC between the model including the temporal fixed effect and null models was ≥2. Then, we compared strength of seasonality by examining the *R*‐squared of the models. It should be noted that *R* ^2^ may not be useful to compare candidate models, e.g., models with and without the temporal fixed effect, because of the nature of the variance partitioning between smoothed and random temporal terms in the GAMs (Knape, 2016). When no smoothed term is included, all temporal variation is attributed to the random intercept term, resulting often in a similar *R*‐squared. However, *R*‐squared can be useful to compare between full models describing different processes, e.g., to compare strength of seasonality in moult vs breeding.

Given the limited temporal span of our data, we also used circular statistics to assess seasonality in bird life cycle events, using the package circular (Agostinelli & Lund, 2023). We calculated mean angle and angular mode to quantify timings of seasonal peaks and mean resultant length to assess strength of seasonal trend (Pewsey et al., 2013) (Appendix S2). Mean angle represents the centre of the seasonal peak, and angular mode indicates the maximum point of the seasonal peak, while mean resultant length is an indicator of the strength of seasonality (Pewsey et al., 2013). We then back converted these angular values to dates, for ease of interpretation. Mean resultant length, calculated as length of the mean resultant vector divided by number of observations, ranges from 0 to 1, with larger values indicating stronger seasonality (Pewsey et al., 2013). We used Rayleigh tests, with the function ‘rayleigh. test’ from the circular package, to test for uniformity in bird life cycle events. In the Rayleigh test, if the *p*‐value is <.05, we reject the null hypothesis that data are uniformly distributed (i.e., in our context, *p* < .05 indicates seasonality; Pewsey et al., 2013).

#### 2.5.2 Correlations between breeding and moult and resource availability

To investigate the relationship between breeding and moulting, fruit tree and arthropod abundance and precipitation, we used generalised linear models (GLMs), assuming a binomial distribution. As birds were surveyed several days per month but arthropods and fruit only once (Appendix S1), we used linear interpolation to achieve daily values of arthropod and fruit abundance that could be matched to the precise dates on which birds were surveyed. To achieve daily values for arthropod and fruiting tree abundance from monthly surveys, we considered the sum across replicates (transects for trees, and trees for arthropods), and then interpolated linearly between monthly datapoints. Daily values of fruit tree and arthropod abundance, alongside precipitation, were included as explanatory variables in the GLMs. We performed model selection using the ‘dredge’ function from the MuMIn package (Bartoń, 2024), selecting models with the lowest Akaike's Information Criterion corrected for small sample size (with ∆AICc < 2; Burnham & Anderson, 2002). All analyses were conducted using RStudio version 2023.3.0.386 in the R computing environment (R Core Team, 2023).

## 3 RESULTS

**FIGURE 1:**
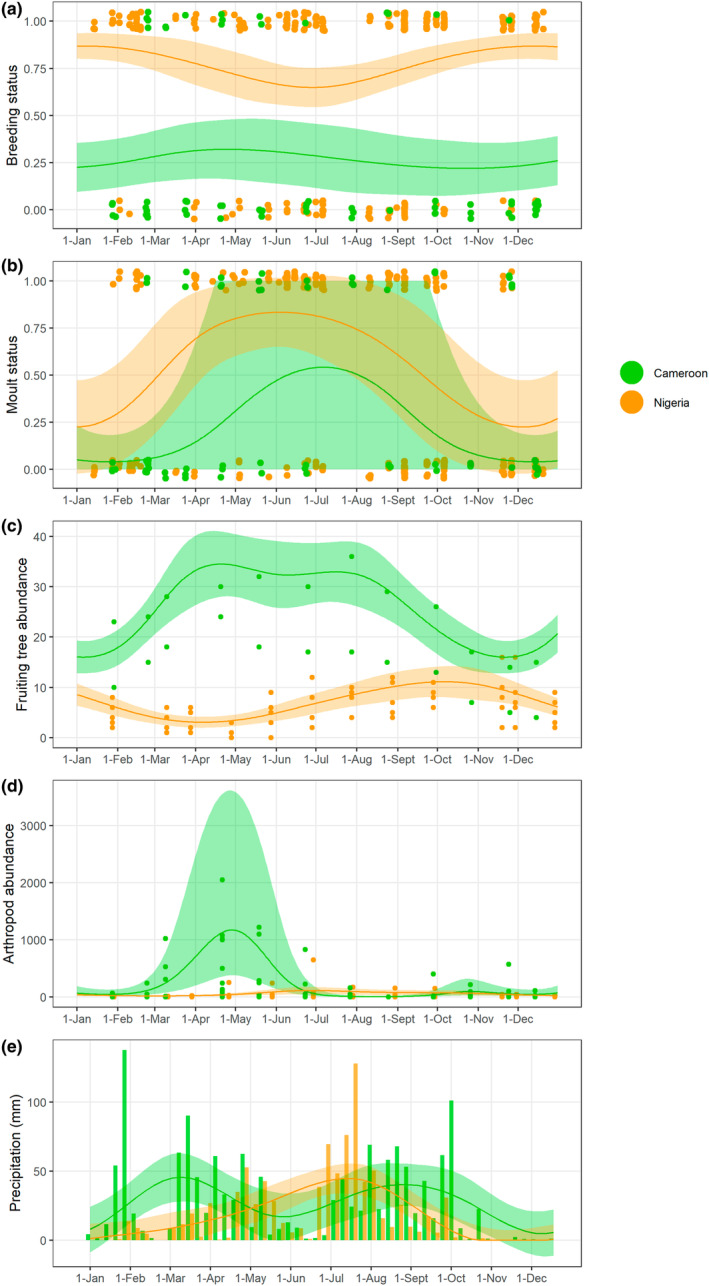
Timing of bulbul breeding (a), primary moult (b), fruiting tree abundance (per 100 m transect) (c) arthropod abundance (per surveyed tree) (d) and precipitation (e) in Cameroon (green) and Nigeria (orange). (a, b) Each dot is an individual bird, which may be breeding (breeding status = 1) or not (breeding status = 0), and/or performing primary moult (moult status = 1) or not (moult status = 0). The continuous lines represent predictions from GAMs with a cyclic cubic spline for day of year and a random effect for week, and shaded areas represent standard error. (c, d) Dots represent counts of fruit or arthropods in each transect (for fruit) or tree (for arthropods). Lines and shaded areas show predictions from GAMs, as above. (e) Weekly precipitation, estimated by the sum of daily accumulated precipitation (mm), is represented by columns, green for Cameroon and orange for Nigeria.

Timing of breeding was seasonal in Nigeria according to the GAMs, with the model including the seasonal term performing significantly better than the random intercept only model (Table 1). Results from circular statistics indicated otherwise, with *p* = .37 in Rayleigh test (Table 1). Probability of breeding was highest in Nigeria in December–January (Figure 1, Figure S3; Table 1). We did not detect a seasonal trend in timing of breeding in Cameroon (Table 1; Figure 1, Figure S3). Primary moult was seasonal in Nigeria and more strongly predicted by date compared with breeding (25% variance explained vs. 5%; Table 1). Moulting peaked in June in Nigeria (Figure 1, Figure S3; Table 1). In Cameroon, there was weak evidence for seasonality in timing of moult (∆AIC = 1.5) and stronger evidence from circular statistics (Table 1), with moult peaking in July but with high uncertainty associated to predictions (Figure 1, Figure S3; Table 1). Overall, therefore, moult was more seasonal than breeding in both sites, and both life cycle events were more seasonal in Nigeria than Cameroon, following predictions (Table 1). Interestingly, moult was similarly timed between the two sites, with the peak in Cameroon occurring ~6‐weeks before the peak in Nigeria.

In lower trophic levels (fruit, arthropods), there was seasonal variation in both sites, following different timing (Table 1; Figure 1, Figures S4 and S5). In Nigeria, fruiting tree abundance was highest in September–October, and lowest in April, while arthropods were most abundant in July, and least abundant in March. In Cameroon, fruiting tree abundance was highest in April (according to GAMs) and August (circular statistics), and lowest in November–December, and strength of seasonality was higher compared with Nigeria. Arthropod abundance spiked dramatically in April–May, mostly due to high abundance of aphids, mealybugs and ants, and was lowest in August (Figure 1, Figure S5). The difference between Nigeria and Cameroon in time of peak fruiting tree abundance was therefore large (~5 months), but smaller for peak arthropod abundance (1.5 months), but in both cases abundance peaked earlier in Cameroon.

In Cameroon there was higher yearly rainfall (1415 mm) than in Nigeria (889 mm), and in both sites rainfall showed strong and distinct seasonal patterns (Figure 1; Table 1). The times of highest rainfall in Nigeria were July–August, and the driest period was November–December. In Cameroon, rainfall appeared to follow a bi‐modal pattern, with high rainfall in March–April and September–October, and the lowest in December–January.

**FIGURE 2:**
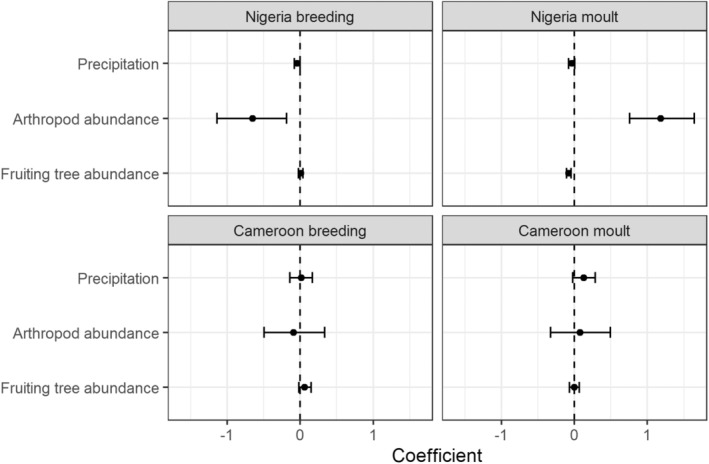
Regression coefficients from full models for the association between fruiting tree abundance, arthropod abundance and precipitation, and bulbul breeding and moult in Cameroon and Nigeria. Points represent estimates obtained from GLMs, and bars represent 95% confidence intervals around predictions.

With regards to correlations between moult and breeding and environmental conditions, in Nigeria, probability of breeding was negatively correlated with arthropod abundance and precipitation, and positively with fruiting tree abundance (Figure 2; Appendix S3). In contrast, in Cameroon, the models containing fruit and precipitation performed similarly well; therefore, breeding was positively associated to higher fruit abundance or higher precipitation (Figure 2; Appendix S3). Probability of primary moult in Nigeria was highest during the period of maximum arthropod abundance, and was negatively correlated to fruiting tree abundance and precipitation. In Cameroon, moult was significantly positively correlated with precipitation, and there was no significant relationship with arthropod or fruit abundance (Figure 2; Appendix S3). Therefore, our expected links between arthropod abundance and breeding, and fruit abundance and moulting were not met at either site.

## 4 DISCUSSION

Common bulbuls showed varying seasonality in breeding and moulting in our study sites, and the specific timing of events was different between sites. Phenology of arthropods and fruit in the environment also varied between sites, with peaks in abundance occurring earlier in Cameroon than Nigeria, as predicted. In bird life cycles, we found stronger seasonality in moult than breeding at both sites, and overall stronger seasonality in Nigeria, both according to expectations. In contrast, the specific timing of bird life cycle events, especially moult, were unexpected. These findings provide additional support to the growing evidence that tropical birds time their life cycles to environmental fluctuations, but that these adjustments may vary over small spatial scales.

In Nigeria, our results suggest a strong seasonal pattern in moult, likely associated to the seasonal rise in arthropods in the environment. Specifically, certain arthropod groups such as Lepidoptera, Hymenoptera and Hemiptera had high abundances coinciding with peak moult timing (Figure S5), suggesting that potentially birds time their moult to specific arthropod food resources. As moult involves not only the replacement of feathers themselves but also the accompanying tissues (sheaths, epidermal structures), birds need to consume sufficient protein during the moulting period (Jenni & Winkler, 2020). Insects contain high protein, and therefore birds may increase insect consumption during moult (Jenni & Winkler, 2020; Pap et al., 2008; Simon et al., 2023). Indeed, birds fed insufficient protein under experimental settings had delayed or slower moult, or produced feathers of lower quality (Jenni & Winkler, 2020; Pap et al., 2008). These findings linking arthropod abundance and moult match recent dietary analyses of bulbuls in Cameroon, which showed that moulting individuals consumed a higher diversity of arthropods than non‐moulting birds, especially Lepidoptera (CJ, CJN & AJW, unpublished data). However, our moult model for Cameroon indicated no associations with arthropods or fruit; while the lack of correlation could be partly a consequence of small sample sizes, it could also reflect differing resource limitationexperienced by bulbuls.

In line with the moult‐arthropod association, breeding in Nigeria was negatively associated with arthropod abundance, indicating that birds may synchronise their moult to peak arthropod abundance, and consequently breed at times when arthropods are less abundant (Class & Moore, 2013). Instead, breeding in Nigeria and Cameroon appeared to be associated with fruiting tree abundance. While it is unlikely that bulbuls feed fruit to their chicks, which in most omnivorous or insectivorous bird species are fed protein‐rich easily digested insects such as caterpillars (Jarrett et al., 2020), it could be that adults consume fruit during the breeding period to increase calory intake. Fruits can constitute high energy foods, consumed for instance by birds prior to migration to increase energy reserves (Bairlein & Gwinner, 1994; Iwajomo et al., 2017). Therefore, breeding at the time of peak fruiting tree abundance could allow maintenance of body conditions while undertaking this energy‐expensive life cycle event. Interestingly, for Cameroon, the model including precipitation performed equally well to the model including fruit at explaining timing of breeding, indicating that breeding could be associated to higher precipitation. Independent of food, associations to precipitation may reflect habitat requirements for nesting or links between precipitation and nest predation (Nwaogu, 2019).

Our results indicate that bulbuls in the Afrotropics show stronger seasonality in moult that in breeding. Additionally, for moult, birds in Nigeria and Cameroon showed close timing, with the peak in probability of primary moult occurring ~1.5 months earlier in Nigeria than in Cameroon. The stronger seasonality in moult and the closer timing of moult between sites follow from each other, as cycles with high amplitude are more robust and respond weakly to fluctuations in environmental cues (Tokuda et al., 2020). The high consistency supports the idea of moult as a more strongly seasonal fixed event, and perhaps as an anchor of the bulbul's annual cycle. In comparatively long‐lived organisms experiencing strong seasonal constraints on the timing of moult, but with multiple breeding opportunities with limited output per year, there might be a weak selection to organise the annual cycle around breeding (Nwaogu et al., 2019). In contrast, there will be a stronger selection to organise the annual cycle around moult. However, the earlier moult in Nigerian birds goes against our expectations, given the findings in Nwaogu and Cresswell (2020), which show later moult at higher latitudes within Nigeria. Based on our study with only two sites, though, we cannot draw conclusions about latitudinally driven patterns as there may be many other factors driving such variation.

If selection acts more strongly on the timing of moult, then seasonality in breeding may simply arise because of seasonal moult, but not because breeding is the event that is timed primarily to specific environmental conditions. This idea is backed up by the coinciding peak timing for primary moult in Nigeria between this study and a previous study from the same site and sites on higher latitudes (Nwaogu et al., 2019; Nwaogu & Cresswell, 2020). This contrasts with temperate‐zone biased suggestions of relatively fixed breeding phenology as an anchor for avian cycles, while moult functions as a more flexible buffer (Helm & Gwinner, 2006). Indeed, we found mixed support for seasonality in timing of breeding in Nigeria, with results from GAMs and circular statistics disagreeing with each other, and in Cameroon, we found no evidence of seasonal breeding.

Overall, our study provides novel evidence of the seasonal yet spatially variable phenology of tropical birds entrained to local environmental seasonality. However, given the limited temporal span of our data, we cannot be certain about the consistency of such patterns between years, and urge longer‐term year‐round studies across latitudinal gradients to tease apart the drivers of such variation. To ensure robust results from our data, we used both GAMs and circular statistics to detect seasonal trends. Importantly, we found that results from GAMs and circular statistics were consistent, with a few exceptions: moult in Nigeria was seasonal according to GAMs but not according to the Rayleigh test, and timing of peak fruiting in Cameroon was April according to GAMs and August according to circular statistics. The former inconsistency may be due to a higher flexibility of the GAM to accommodate seasonal patterns, and to the presence of the temporal random effect in the GAM that may absorb some of the noise in the data. The latter is likely due to the seasonal plateau in fruit abundance in Cameroon between April and August (Figure 1).

In conclusion, our results suggest that over small spatial scales, timing of resources may vary considerably, leading to different life cycle timing in birds, but with differential effects for life cycle stages. Understanding bird phenology is important as it can reveal vulnerabilities of species in the face of continued land‐use and climate change (Cohen et al., 2018; Fotso, 1996; Helm et al., 2013; Samplonius et al., 2016; Thomas et al., 2001). Incorporating a temporal dimension to conservation efforts, for instance in the seasonal control of burning in the Afrotropics, could encourage win‐win scenarios for humans and wildlife in transformed landscapes (Fotso, 1996). With our knowledge of tropical bird life cycles lagging behind that of temperate counterparts, our study reveals the importance of considering local context in the study of tropical birds, and provides additional evidence of the strong seasonal nature of tropical environments.

## AUTHOR CONTRIBUTIONS

**Crinan Jarrett:** Conceptualization (equal); formal analysis (lead); investigation (equal); methodology (equal); project administration (equal); visualization (lead); writing – original draft (lead); writing – review and editing (equal). **Ojodomo Godday Simon:** Data curation (equal); investigation (equal); methodology (equal); writing – review and editing (equal). **Christian N. Tchana:** Data curation (equal); investigation (equal); methodology (equal); writing – review and editing (equal). **Thaddeus Apezan Pev:** Data curation (equal); investigation (equal); methodology (equal); writing – review and editing (equal). **Michelle Fany Meigang Kamkeng:** Data curation (equal); investigation (equal); methodology (equal); writing – review and editing (equal). **Alain Christel Wandji:** Data curation (equal); investigation (equal); methodology (equal); writing – review and editing (equal). **Shiiwua A. Manu:** Project administration (equal); supervision (equal); writing – review and editing (equal). **Mélanie Adèle Tchoumbou:** Methodology (equal); project administration (equal); supervision (equal); writing – review and editing (equal). **Barbara Helm:** Conceptualization (equal); formal analysis (equal); funding acquisition (equal); investigation (equal); methodology (equal); project administration (equal); supervision (equal); writing – review and editing (equal). **Luke L. Powell:** Conceptualization (equal); funding acquisition (equal); methodology (equal); project administration (equal); writing – review and editing (equal). **Chima J. Nwaogu:** Conceptualization (equal); formal analysis (equal); investigation (equal); methodology (equal); project administration (equal); supervision (equal); writing – review and editing (equal).

## CONFLICT OF INTEREST STATEMENT

The authors declare that they have no conflicts of interest.

## Supporting information


Data S1.

## Data Availability

Data are archived in Figshare (10.6084/m9.figshare.25188020).
