# Supplementary material for: Differences in phenology across three trophic levels between two Afrotropical sites separated by four degrees latitude
Source: Ecol Evol. 2024 Sep 11;14(9):e70274. doi: 10.1002/ece3.70274 (PMC11391021; doi:10.1002/ece3.70274)
Supplement: Supplementary file 1 — Data S1. [file ECE3-14-e70274-s001.docx]

**SUPPLEMENTARY MATERIALS**

**Differences in phenology across three trophic levels between two Afrotropical sites separated by four degrees latitude**

**APPENDIX S1: Additional information on study sites and survey dates**

**
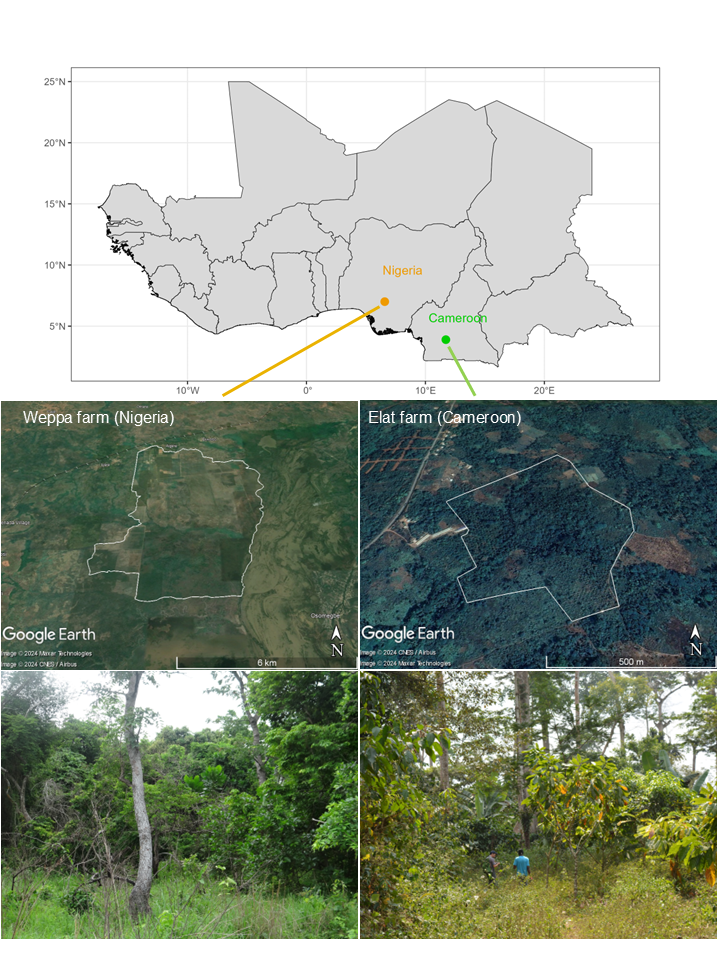
**

**Figure S1.** Location of the two study sites, Weppa farm (Nigeria) and Elat farm (Cameroon), in West Africa, farm boundary and example of habitat.

| **Table S1.** Dates of fruit tree and arthropod field surveys in Cameroon and Nigeria, with a column showing the temporal random effect (week) included in models. | | | | | |
| --- | --- | --- | --- | --- | --- |
| **Site** | **Visit** | **Fruit tree survey date** | **Arthropod sampling date** | **Month** | **Week (random effect in model)** |
| Cameroon | 1 | 20.04.2021 | 21.04.2021 | 4 | 16 |
| Cameroon | 2 | 19.05.2021 | 19.05.2021 | 5 | 20 |
| Cameroon | 3 | 25.06.2021 | 23.06.2021 | 6 | 25 |
| Cameroon | 4 | 28.07.2021 | 27.07.2021 | 7 | 30 |
| Cameroon | 5 | 24.08.2021 | 25.08.2021 | 8 | 34 |
| Cameroon | 6 | 30.09.2021 | 28.09.2021 | 9 | 39 |
| Cameroon | 7 | 27.10.2021 | 26.10.2021 | 10 | 43 |
| Cameroon | 8 | 25.11.2021 | 24.11.2021 | 11 | 47 |
| Cameroon | 9 | 15.12.2021 | 14.12.2021 | 12 | 50 |
| Cameroon | 10 | 29.01.2022 | 28.01.2022 | 1 | 4 |
| Cameroon | 11 | 24.02.2022 | 23.02.2022 | 2 | 8 |
| Cameroon | 12 | 10.03.2022 | 09.03.2022 | 3 | 10 |
| Nigeria | 1 | 28.04.2021 | 26.04.2021 | 4 | 17 |
| Nigeria | 2 | 28.05.2021 | 29.05.2021 | 5 | 21 |
| Nigeria | 3 | 28.06.2021 | 29.06.2021 | 6 | 26 |
| Nigeria | 4 | 28.07.2021 | 29.07.2021 | 7 | 30 |
| Nigeria | 5 | 28.08.2021 | 30.08.2021 | 8 | 34 |
| Nigeria | 6 | 28.09.2021 | 29.09.2021 | 9 | 39 |
| Nigeria | 7 | 19.11.2021 | 19.11.2021 | 11 | 46 |
| Nigeria | 8 | 29.11.2021 | 30.11.2021 | 11 | 48 |
| Nigeria | 9 | 29.12.2021 | 29.12.2021 | 12 | 52 |
| Nigeria | 10 | 28.01.2022 | 29.01.2022 | 1 | 4 |
| Nigeria | 11 | 10.03.2022 | 11.03.2022 | 3 | 10 |
| Nigeria | 12 | 28.03.2022 | 29.03.2022 | 3 | 12 |


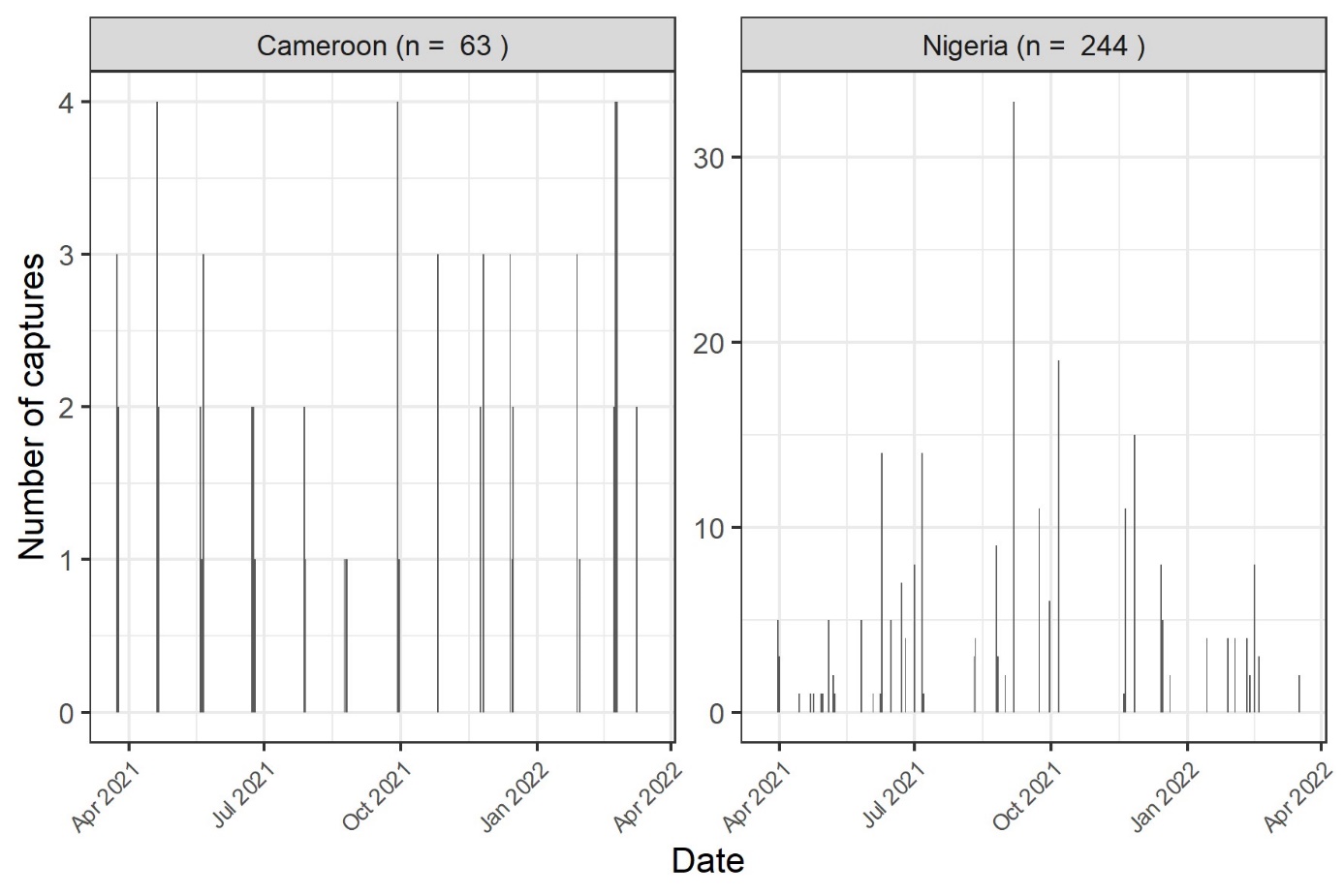


**Figure S2.** Number of captures of Common Bulbul (*Pycnonotus barbatus*) according to date in Cameroon and Nigeria. Total captures across the year are indicated in the facet titles.

**APPENDIX S2: Data exploration using circular statistics**

For bird life-history data, mean angle and angular mode indicated that breeding in Nigeria peaked in early January, and moult in mid to late June. For Cameroon, breeding peaked in April-May, and moult in mid-July. These summary statistics from circular statistics match closely the predicted peak timings from the General Additive Models (GAMs; see Main Text). Rayleigh tests indicated that in Nigeria breeding was non-seasonal (p=0.37) but moult was seasonal (p<0.001). In Cameroon, breeding was not seasonal (p=0.11) and moult was seasonal (p=0.006). Broadly, these statistics match results from the GAMs, with the exception of breeding in Nigeria which according to the GAMs did show seasonal patterns.

In Nigeria, in two cases (Oct-Nov 2021 and Feb-March 2022), fruit tree and arthropod surveys were not spaced evenly by one month (Table S1). For the circular statistics, to make the data comparable to Cameroon and evenly spaced out by month, we interpolated from the existing data points (spaced out by ~6 weeks) to achieve monthly values (Fig. S4-S5).


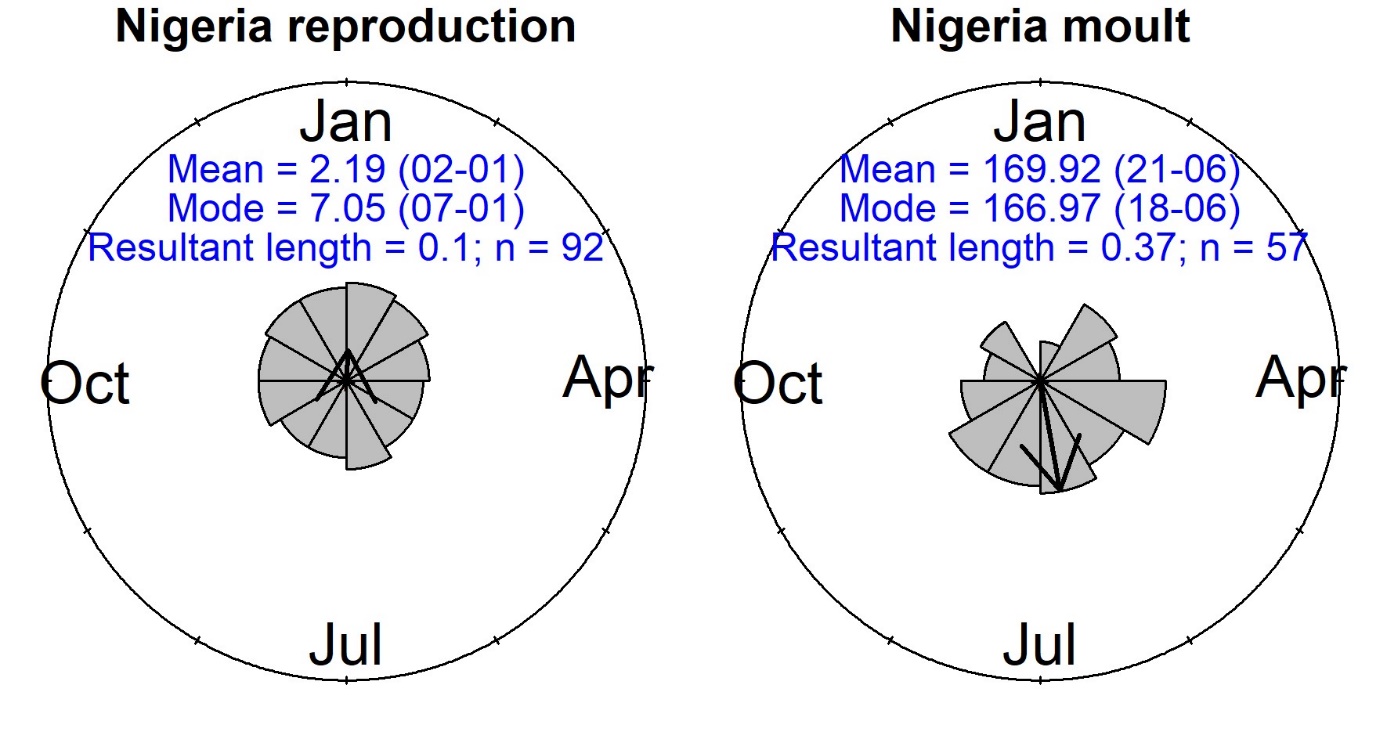


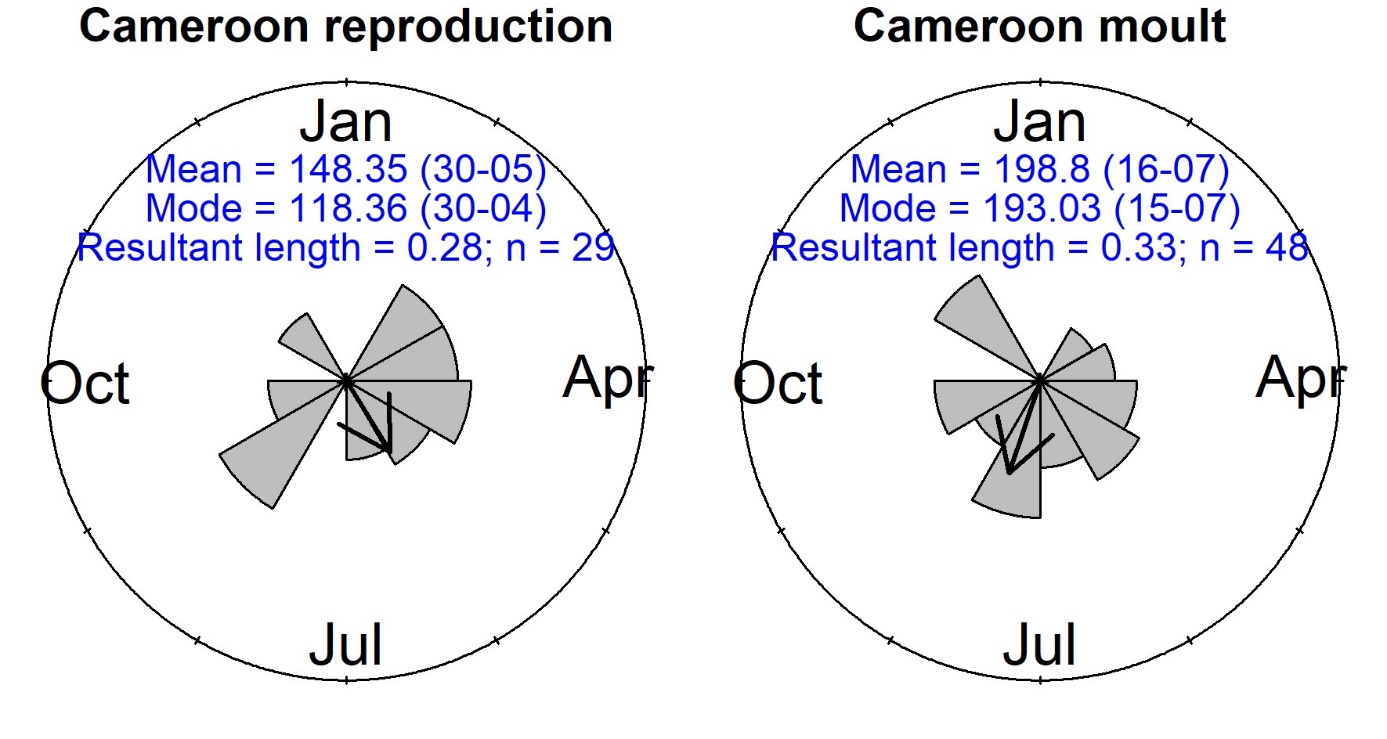


**Figure S3.** Annual distribution of proportion of total individuals breeding and moulting in Nigeria and Cameroon around the study year. Rose diagrams show histograms at monthly resolution, and the text includes mean angle and angular mode of reproductive and moult phenology, and mean resultant length (length of the mean resultant vector divided by number of observations - an indicator of amplitude of phenology). Mean angle, angular mode and resultant length were computed following Pewsey et al. (2013), and we back-converted mean angle and angular mode to date (displayed in brackets in format day-month). Total number of individual birds actively breeding or moulting throughout the year is indicated as n = number of individuals.


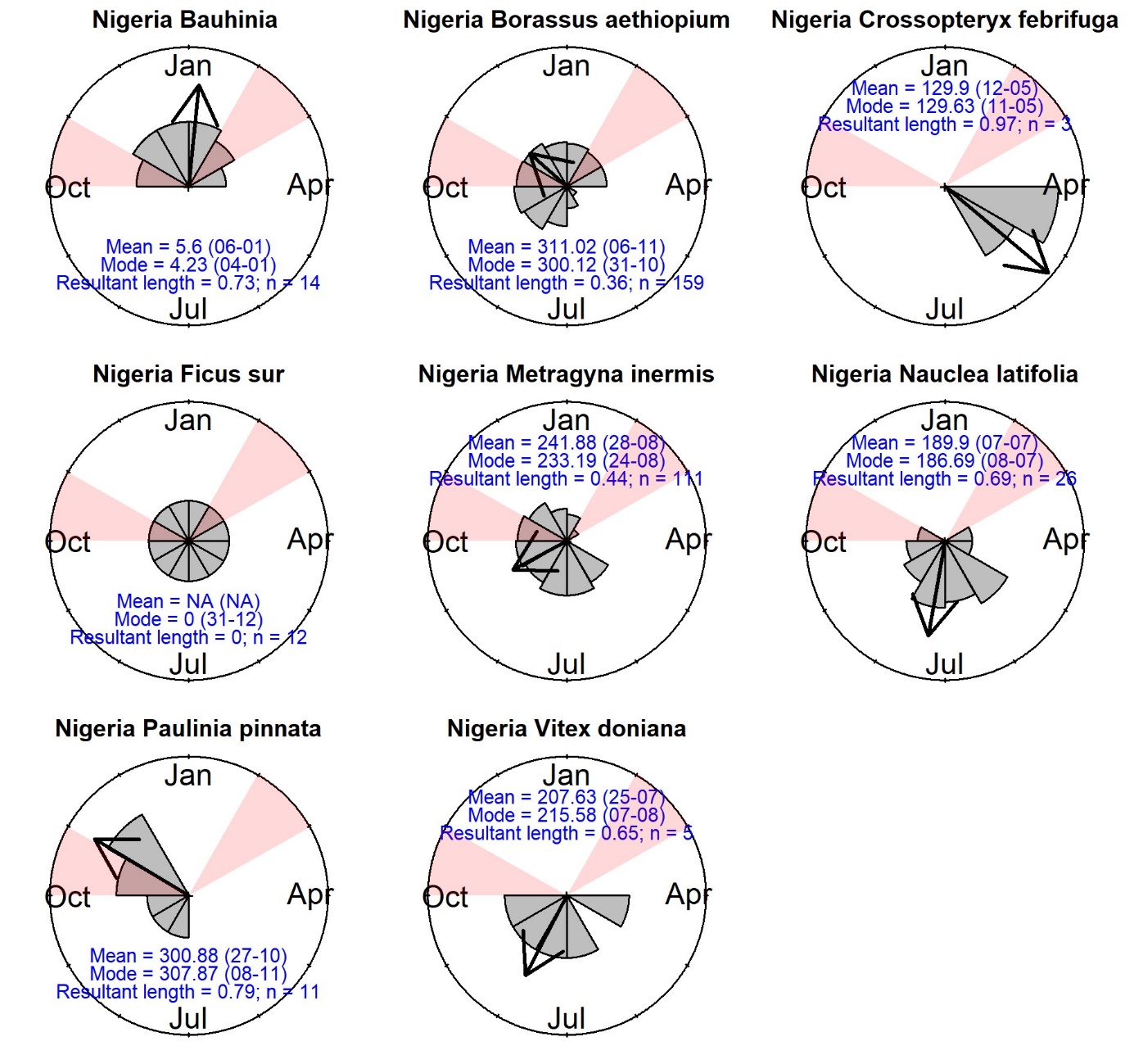


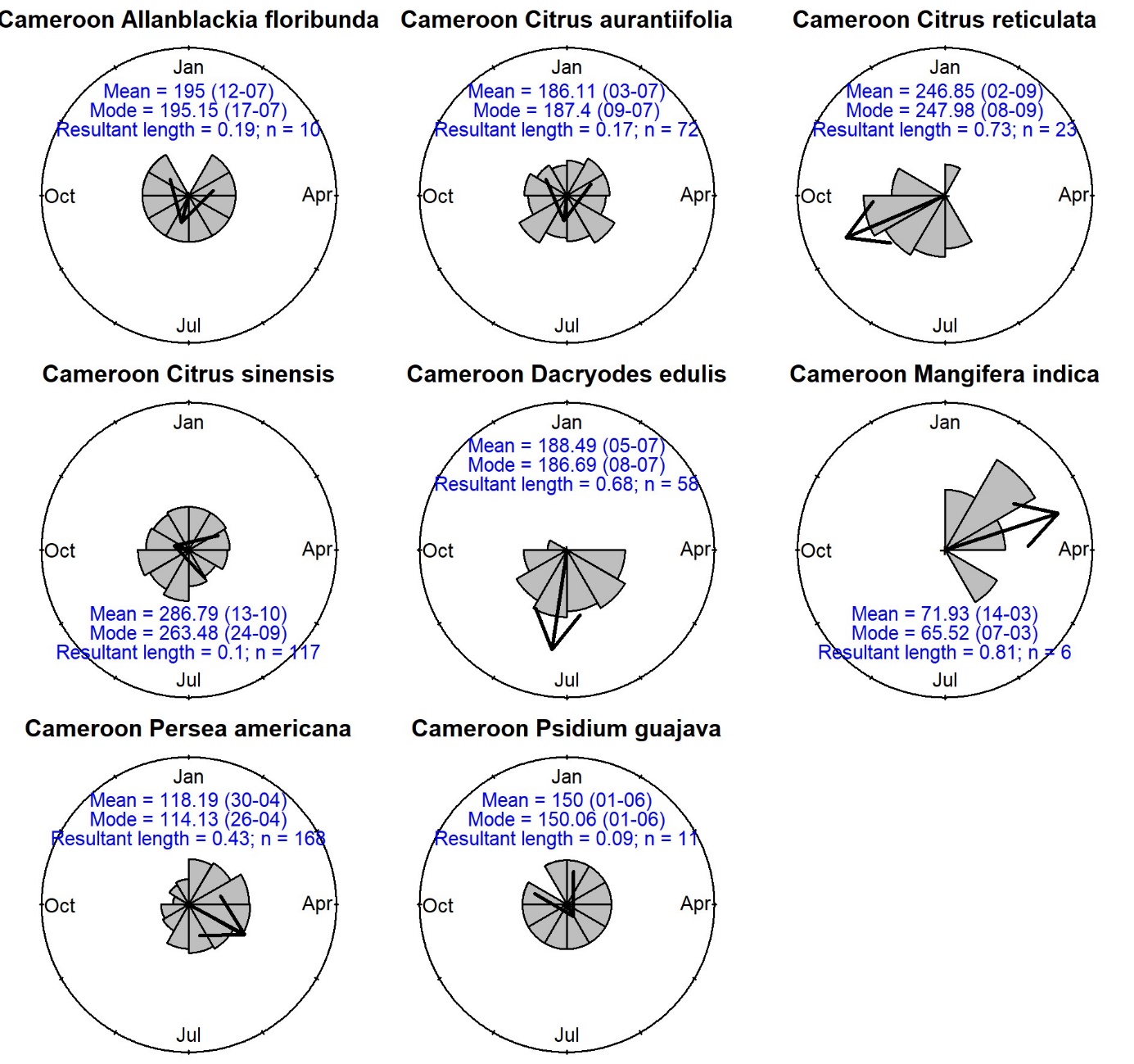


**Figure S4.** Annual distribution of fruiting tree abundance in Nigeria and Cameroon around the study year. Rose diagrams show histograms at monthly resolution, and the text includes mean angle and angular mode of fruiting phenology, and resultant length (an indicator of amplitude of phenology). The abundance of trees is the sum across transects (n=5 in Nigeria, n=2 in Cameroon). Mean angle, angular mode and mean resultant length were computed following Pewsey et al. (2013), and we back-converted mean angle and angular mode to date (displayed in brackets in format day-month). Total number of trees counted of each species throughout the year is indicated as n = number of trees. In Nigeria, for the months of October and February (highlighted in pink), the values displayed are interpolated from the available data, that corresponded to later sampling dates.


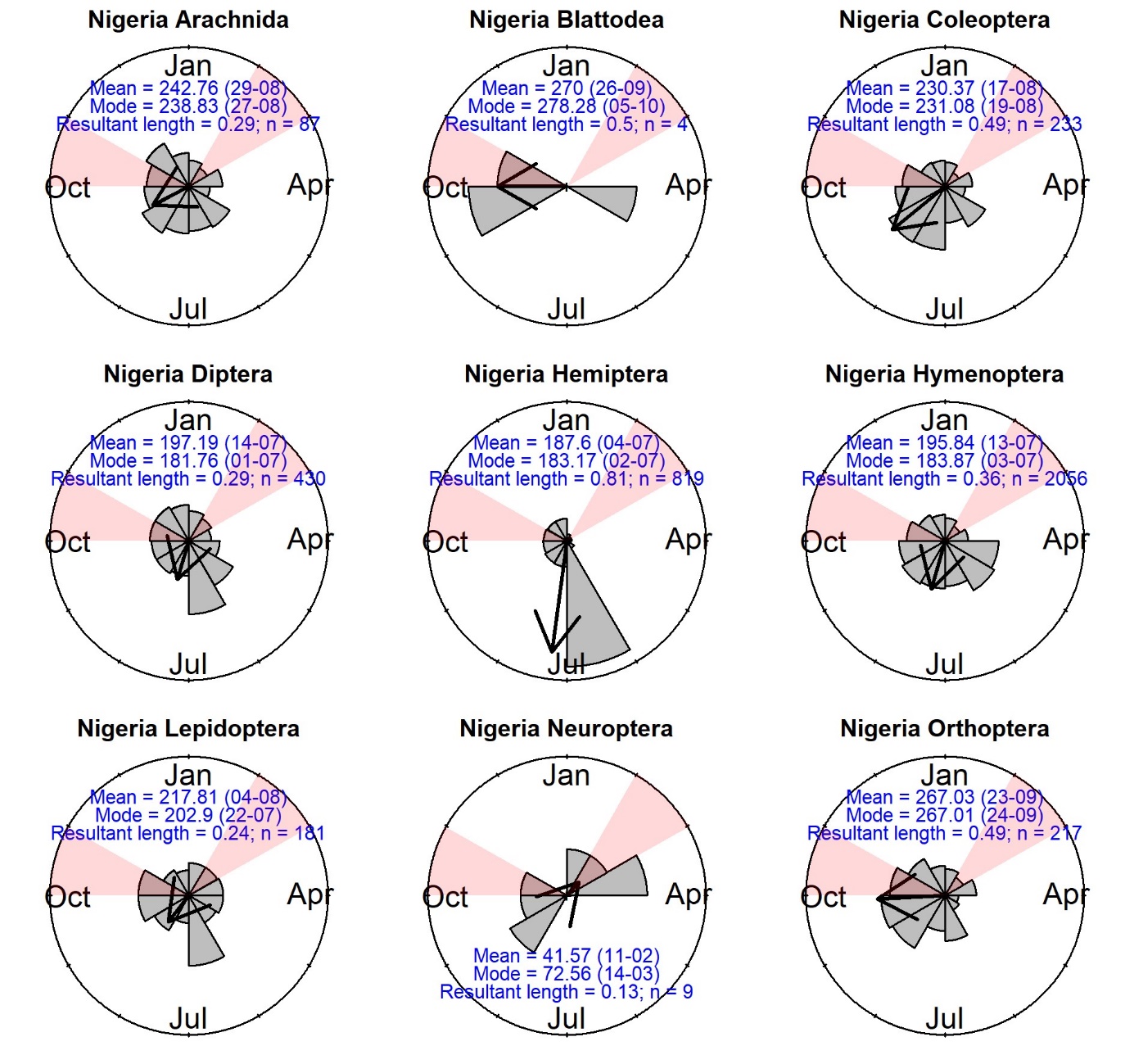


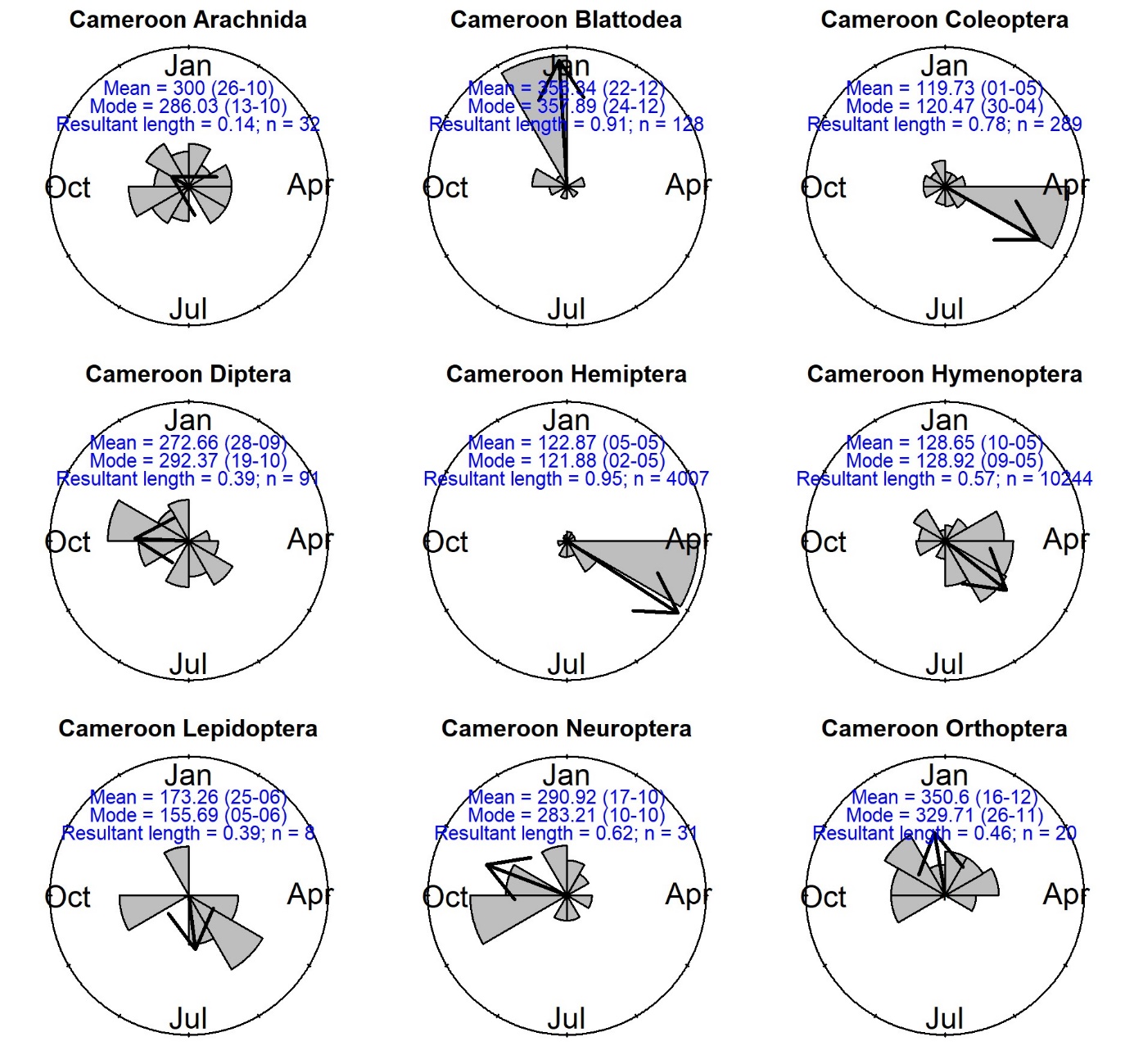


**Figure S5.** Annual distribution of arthropod order abundance in Nigeria and Cameroon around the study year. Rose diagrams show histograms at monthly resolution, and the text includes mean angle and angular mode of arthropod phenology, and resultant length (an indicator of amplitude of phenology). Mean angle, angular mode and mean resultant length were computed following Pewsey et al. (2013), and we back-converted mean angle and angular mode to date (displayed in brackets in format day-month). Total number of arthropods counted of each order throughout the year is indicated as n = number of trees. In Nigeria, for the months of October and February (highlighted in pink), the values displayed are interpolated from the available data, that corresponded to later sampling dates.

**APPENDIX S3: Model selection for GLMs**

| **Table S2.** Models used to describe relationship between breeding and moulting and fruiting tree abundance, arthropod abundance (log transformed) and precipitation. Models are organised so that the one with the lowest AIC value is at the top, and we present only the best fit models (∆AIC < 2). | | | | |
| --- | --- | --- | --- | --- |
| **Site** | **Response variable** | **Explanatory variables** | **AIC** | **∆AIC** |
| Nigeria | Breeding | log (arthropod abundance) + precipitation | 260.1 | 0.0 |
|  |  | fruiting tree abundance + log (arthropod abundance) + precipitation | 262.0 | 1.9 |
|  | Moulting | fruiting tree abundance + log (arthropod abundance) + precipitation | 301.8 | 0.0 |
|  |  | fruiting tree abundance + log (arthropod abundance) | 303 | 1.2 |
|  |  |  |  |  |
| Cameroon | Breeding | fruiting tree abundance | 73.6 | 0.0 |
|  |  | precipitation | 75.4 | 1.8 |
|  | Moulting | precipitation | 81.5 | 0.0 |

**References**

Agostinelli, C., & Lund, U. (2023). *R package “circular”: Circular Statistics* (0.5-0). https://cran.r-project.org/package=circular

Pewsey, A., Neuhäuser, M., & Ruxton, G. D. (2013). *Circular Statistics in R*. Oxford University Press.
